# Plants of the family Lamiaceae as a source of therapeutic agents against *Acanthamoeba* infections

**DOI:** 10.1590/0074-02760240171

**Published:** 2024-11-11

**Authors:** Martin Mrva, Lucia Malíková, Mária Garajová

**Affiliations:** 1 Comenius University in Bratislava, Faculty of Natural Sciences, Department of Zoology, Bratislava, Slovakia; 2 Modra, Slovakia

**Keywords:** Acanthamoeba, Lamiaceae, plant extracts, essential oils, amoebicidal effect

## Abstract

**BACKGROUND:**

Acanthamoebae are causative agents of severe and complicated human infections without a standard effective therapy to date. Therefore, the research is focused on the development of new amoebicidal drugs based on the natural products. Plants of the family Lamiaceae are typical with several phenolic secondary metabolites that make them interesting in medical point of view.

**OBJECTIVE:**

In this review, we concentrate on anti-*Acanthamoeba* activities of plant extracts, essential oils, and phytochemicals of Lamiaceae in the published literature.

**FINDINGS:**

A total of 13 articles in the research field were found. Totally, 16 plant species belonging to family Lamiaceae were studied against trophozoites and cysts of *Acanthamoeba* in *in vitro* conditions. Low toxicity of the Lamiaceae plant extracts to tissue cultures enhances their possible potential for clinical use. The research demonstrated promising trophocidal and cysticidal effects against acanthamoebae. Further research is needed with inclusion of more clinical isolates and *in vivo* studies.

**MAIN CONCLUSION:**

Reviewing the related literature highlights the promising amoebicidal activities of plant extracts, essential oils and bioactive compounds of family Lamiaceae. Identifying the active components could lead to production new effective and well-tolerated drugs for the *Acanthamoeba* infections treatment.

Acanthamoebae are free living amphizoic amoebae occuring commonly in soil, fresh water and anthropogenic habitats.^1^ In their life cycle, besides the amoeboid trophozoite, a highly resistant cyst with double-layered wall is found.^2^ ^,^ ^3^ ^,^ ^4^ The modern classification model based on 18S rDNA sequences identified 23 genotypes (T1 - T23), up to date.^5^ The highest number of human infections is associated with T4 genotype isolates.^6^ Even the environmental strains belonging to the T4 genotype are typical frequently with virulence factors, such as osmotolerance, thermotolerance and secretion of extracellular proteases.^7^

Pathogenic strains are causative agents of severe and complicated human infections: granulomatous amoebic encephalitis (GAE), disseminated, cutaneous, nasopharyngeal infections, and *Acanthamoeba* keratitis (AK). GAE is a rare chronic infection of the central nervous system (CNS) with poor prognosis, which affects mainly immunodeficient individuals.^8^ ^,^ ^9^ AK is a painful progressive infection of the eye cornea mainly in immunocompetent contact lenses wearers.^3^ If the infection is not treated at an early stage, extensive eye damage and vision loss may occur.^10^ Potential risk present also the interactions of acanthamoebae with pathogenic bacteria and viruses which can multiply in their cytoplasm and subsequently spread to the environment^11^ while increasing *Acanthamoeba* pathogenicity^12^ ^,^ ^13^ and resistance to amoebicidal drugs.^14^ These features possibly can be result of the horizontal gene transfer which recently has been proven between *Acanthamoeba* and amoeba-resisting microorganisms (ARMs).^15^ However, further research is needed to elucidate this process.

To date no standard effective therapy against *Acanthamoeba* infections have been developed. For the treatment of GAE, diverse drugs have been applied in combinations with various effects: antibiotics (macrolides), antifungals (fluconazole, itraconazole, amphotericin B), pentamidine isethionate, sulfadiazine, flucytosine, trimethoprim-sulfamethoxazole, miltefosine.^16^ ^,^ ^17^ However, the treatment of GAE, cutaneous and disseminated infections are only rarely successful. Treatment of AK is based mainly on polyhexamethylene-biguanide, diamidine (propamidine, hexamidine) and chlorhexidine with antifungal drugs.^18^ Miltefosine in combinations with these compounds was experimentally tested, as well.^19^ The treatment of AK must be very intensive with frequent topical drug application.^20^ However, recurrence or weak response to treatment may lead to keratoplasty or even to eye enucleation.^21^

Since the number of diagnosed cases of infections is increasing worldwide, it is necessary to develop more effective amoebicidal drugs.^22^ ^,^ ^23^ ^,^ ^24^ Standard methods of drugs screening based on *in vitro* experiments can be combined with virtual screening methods using the computational approach and identifying effective chemical groups.^25^ A new strategy for alternative and effective treatment of *Acanthamoeba* infections may represent plant extracts rich in biologically active ingredients.^26^ ^,^ ^27^ Natural products derived from plants play an important role in the control of various diseases caused by parasitic protists at present. In the treatment of malaria caused by *Plasmodium falciparum* artemisinin and quinine are standardly used.^28^ ^,^ ^29^ Other biologically active compounds from plants or plant extracts have been successfully tested against *Plasmodium* spp., *Entamoeba histolytica*, *Cryptosporidium parvum*, *Giardia intestinalis*, *Leishmania* spp., *Toxoplasma gondii*, *Trichomonas vaginalis*, *Trypanosoma* spp.^30^ ^,^ ^31^ ^,^ ^32^ A high quantity of active compounds from plants contains propolis (bee glue) which also exhibited anti-protozoal activity.^33^

The family Lamiaceae is one of the most diverse families within the flowering plants with more than 7500 species distributed worldwide.^34^ Lamiaceae plants are typical with several phenolic secondary metabolites that make them interesting in medical point of view.^35^ ^,^ ^36^ ^,^ ^37^ The most typical is rosmarinic acid (RA) which exhibits number of biological activities as antioxidant, anti-inflammatory and antimicrobial effects.^38^ Several works on Lamiaceae as a source of new amoebicidal agents against pathogenic *Acanthamoeba* strains were published, as well. This review focuses on the present knowledge on amoebicidal activities of Lamiaceae plant extracts and essential oils.

Amoebicidal activities of Lamiaceae

It has been proven that essential oils obtained from the leaves and flowers of *Satureja cuneifolia* and *Satureja montana* inhibit *in vitro* the proliferation of medically important pathogenic bacteria resistant to antibiotics, and the fungicidal activity of the oils has also been proven.^39^ Carvacrol has been identified as the main bioactive component in the more effective oil of *S. montana.* ^40^ The methanolic extract of *S. cuneifolia* showed good elimination abilities against *Acanthamoeba castellanii* trophozoites and cysts (Table I). The 100% trophocidal effect was achieved after 24 h at a concentration of 32 and 16 mg/mL. A concentration of 32 mg/mL had a cysticidal effect, after 72 h of the experiment 53.7% of vital cysts remained.^41^

**TABLE I:** Anti-*Acanthamoeba* activities of extracts from plants of the family Lamiaceae

Plant species	Extract	Amoeba strain	Strain source	Amoebicidal activity	Reference
*Melissa officinalis*	Methanol	*A. castellanii*	clinical	72 h - at 32 mg/mL 44.3% of trophozoites and 30.0% of the cysts killed	^41^
*Origanum laevigatum*	Methanol	*A. castellanii*	clinical	72 h - at 16 mg/mL no viable trophozoites 72 h - at 32 mg/mL 47.7% of the cysts killed	^49^
*Origanum syriacum*	Methanol	*A. castellanii*	clinical	6 h - at 16 mg/mL no viable trophozoites 24 h - at 32 mg/mL no viable cysts	^49^
*Salvia caespitosa*	Methanol	*A. castellanii* 1BU	clinical	72 h - at 8 mg/mL no viable trophozoites, at 16 mg/mL no viable cysts	^43^
*Salvia staminea*	Methanol	*A. castellanii* 1BU	clinical	72 h - at 1 mg/mL no viable trophozoites, at 8 mg/mL no viable cysts	^43^
*Satureja cuneifolia*	Methanol	*A. castellanii*	clinical	24 h - 16 mg/mL no viable trophozoites 72 h - at 32 mg/mL 46.3% of the cysts killed	^41^
*Scutellaria baicalensis*	Aqueous	*A. royreba* *A. castellanii* *A. polyphaga*	Not stated	50 mg/mL - strong trophocidal effect against *A. royreba*, less effective against *A. castellanii* and *A. polyphaga*	^55^
*Teucrium chamaedrys*	Methanol	*A. castellanii*	clinical	48 h - at 16 mg/mL no viable trophozoites 72 h - at 32 mg/mL 30.3% of the cysts killed	^50^
*Teucrium polium*	Methanol	*A. castellanii*	clinical	48 h - at 32 mg/mL no viable trophozoites 72 h - at 32 mg/mL 19.3% of the cysts killed	^50^
*Teucrium ramosissimum*	Hexane, ethyl acetate, acetone, ethanol	*A. terricola* Neff^*^	environmental	72 h - 144 h IC_50_ against trophozoites > 100 µg/mL	^51^
*Thymus capitatus*	Ethanol-aqueous	*A. terricola* Neff^*^	environmental	96 h - IC_50_ against trophozoites 21.51 µg/mL	^47^
*Thymus sipyleus*	Methanol	*A. castellanii* 1BU	clinical	72 h - at 1 mg/mL no viable trophozoites, at 4 mg/mL no viable cysts	^46^

^*^Asterisk marks the strain previously published as Neffʼs strain of *Acanthamoeba castellanii* which was transferred recently to the species *Acanthamoeba terricola* by Corsaro et al.^59^

Plant extracts from several species of the genus *Salvia* have demonstrated antiprotozoal (*Trypanosoma brucei*, *E. histolytica*, *G. intestinalis*), antibacterial and antineoplastic activity.^42^ A significant anti-proliferative effect on *A. castellanii* trophozoites and cysts was achieved in the presence of the methanol extract of the species of sage *Salvia staminea* (Table I). The extract concentration of 16 mg/mL after an exposure time of 48 h had a 100% lethal effect on the cysts and, moreover, it was not toxic to the corneal epithelial cell culture. Even at 8 mg/mL after 72 h, no vital cysts were also observed. The extract from the sage of another species, *Salvia caespitosa* demonstrated weaker, but also significant trophocidal and cysticidal effect.^43^ Abietane diterpenoids extracted and isolated from the roots of a related species *Salvia sclarea* showed interesting amoebicidal activity against *A. castellanii*. The highest trophocidal activity exhibited ferruginol with 50% inhibitory concentration IC_50_ of 0.005 mg/mL followed by aethiopinone and 1-oxo-aethiopinone, both with IC_50_ of 0.05 mg/mL, after 72 h^44^ (Table II).

Further species exhibiting anti-*Acanthamoeba* activity belong to the genus *Thymus*. Thyme essential oils are typical with high content of phenolic compounds ― thymol and carvacrol, and terpene alcohols (*e.g.*, terpineol, geraniol).^45^ In the presence of the methanolic extract of the Turkish thyme species *Thymus sipyleus*, complete eradication of *A. castellanii* trophozoites was achieved after the first hour of incubation at a concentration of 32 mg/mL. This concentration was also 100% cysticidal already after 12 h. At the same time, this dose was not toxic to rabbit corneal cell culture through the whole experiment^46^ (Table I). Very high amoebicidal activity exhibited the essential oil from *Thymus capitatus* with 50% inhibitory concentration IC_50_ of 2.73 µg/mL after 96 h in comparison with ethanol-aqueous plant extract with IC_50_ of 21.51 µg/mL after 96 h. In the essential oil, carvacrol and *p*-cymene have been identified as the most important compounds active against *Acanthamoeba terricola* trophozoites^47^ (Table II).

**TABLE II:** Anti-*Acanthamoeba* activities of the essential oils and bioactive compounds from plants of the family Lamiaceae

Plant species	Most important compounds	Amoeba strain	Strain source	Amoebicidal activity	Reference
*Melissa officinalis*	Not stated	*A. castellanii*	clinical	24 h - at 0.625 µg/mL no viable trophozoites 48 h - at 40 µg/mL no viable cysts	^52^
*Mentha piperita*	Not stated	*Acanthamoeba* sp.	Not stated	24 h - at 30% concentration no viable trophozoites	^53^
*Mentha piperita*	Not stated	*A. castellanii*	clinical	24 h - at 2.5 µg/mL no viable trophozoites 72 h - at 40 µg/mL no viable cysts	^52^
*Ocimum basilicum*	Not stated	*A. castellanii*	clinical	24 h - at 5 µg/mL no viable trophozoites 72 h - at 40 µg/mL 63.3% of the cysts killed	^52^
*Rosmarinus officinalis*	1.8-cineole 25.23%, α-pinene 18.81%, camphor 15.26%, camphene 10.82%, borneol 2.87%, caryophyllene 1.91%	*A. polyphaga* ApUP	clinical	144 h - at 100 µg/mL 50% inhibition of trophozoites	^54^
*Salvia sclarea*	ferruginol, salvipisone, aethiopinone, 1-oxo-aethiopinone	*A. castellanii* 309	environmental	72 h - at 0.1 mg/mL reduction of trophozoites: 71.9% by ferruginol, 38.8% by salvipisone, 56.3% by aethiopinone, 55,3% by 1-oxo-aethiopinone	^44^
*Scutellaria baicalensis*	baicalin, baicalein, wogonoside, oroxylin A	*A. castellanii* ATCC 30234, *A. polyphaga* ATCC 30461	Clinical + isolated from yeast culture	4 h Oroxylin A and wogonoside - 0.4 and 0.5 log reduction in trophozoites viability, respectively. 4 h Combination of baicalein and oroxylin A - 0.50-0.65 log reduction in trophozoites viability.	^56^
*Teucrium ramosissimum*	δ-cadinol 18.70%, δ-cadinene 18.63%, β-eudesmol 12.13%	*A. terricola* Neff^*^	environmental	72 h - IC_50_ against trophozoites 25.73 µg/mL	^51^
*Thymus capitatus*	carvacrol 81.53%, *p*-cymene 6.10%, γ-terpinene 2.64%, caryophyllene 2.66%	*A. terricola* Neff^*^	environmental	96 h - IC_50_ against trophozoites 2.73 µg/mL	^47^

^*^Asterisk marks the strain previously published as Neffʼs strain of *Acanthamoeba castellanii* which was transferred recently to the species *Acanthamoeba terricola* by Corsaro et al.^59^

Many species of plants belonging to the genus *Origanum* have medicinal value. Syrian marjoram (*Origanum syriacum*) as a spice has antiseptic and anti-inflammatory properties. From its essential oil, the main bio-components thymol and carvacrol were isolated. Carvacrol is a phenolic phytochemical responsible for its burning aroma. The volatile phenolic oil obtained from this plant species has shown antibacterial, antifungal, antioxidant and natural preservative properties.^48^ The potential amoebicidal effect of *O. syriacum* extract on *A. castellanii* was therefore investigated and the result was the determination of its trophocidal and cysticidal activity. Elimination of both *Acanthamoeba* stages was achieved at a concentration of 32 mg/mL after 24 h. On the other hand, the methanolic extract of *Origanum laevigatum* had distinctly a weaker effect, as more than 47% of the cysts remained viable even in the highest tested concentration^49^ (Table I).

Similar results were obtained with other plant species: *Teucrium chamaedrys* and *Teucrium polium*, whose extracts were amoebicidal and effective against trophozoites of *A. castellanii*, but the cysts remained viable even at a concentration of 32 mg/mL^50^ (Table I). The essential oil from related species *Teucrium ramosissimum* with main components δ-cadinol, δ-cadinene and β-eudesmol demonstrated 50% inhibitory concentreation IC_50_ of 25.73 µg/mL after 72 h against *A. terricola* trophozoites. The plant extracts (prepared with solvents hexane, ethyl acetate, acetone and ethanol) were much weaker with IC_50_ of > 100 µg/mL^51^ (Table II).

A weaker effect was demonstrated during the incubation of amoebae with an extract from lemon balm *Melissa officinalis*. Its concentration of 32 mg/mL was not sufficient to kill all trophozoites of *A. castellanii* culture even after 72 h of exposure.^41^ However, the essential oil of *M. officinalis* showed much better results. The elimination of 100% trophozoites in the experiment was detected at 0.625 µg/mL already after 24 h and 100% lethal effect on cysts of *A. castellanii* was observed at 40 µg/mL after 48 h^52^ (Table II).

Preliminary screening of trophocidal activity of *Mentha piperita* essential oil at 30% concentration showed 100% elimination of *Acanthamoeba* sp. after 24 h.^53^ In further detailed study Ergüden et al.^52^ noted elimination of 100% of trophozoites by the essential oil of *M. piperita* at 2.5 µg/mL already after 24 h. A 100% lethal effect on cysts of *A. castellanii* was reached at 40 µg/mL after 72 h (Table II).

Weaker results has given the essential oil of *Ocimum basilicum* which showed 63.3% lethal effect on cysts of *A. castellanii* at 40 µg/mL after 72 h and all trophozoites in the experiment were eliminated at 5 µg/mL already after 24 h.^52^

Manifestation of the amoebicidal activity of rosemary *Rosmarinus officinalis* essential oil included morphological changes in trophozoites with precipitates and autophagic vesicles in the cytoplasm. The main detected essential oil components were 1.8-cineole, α-pinene, camphor and camphene. Amoebicidal effect of the essential oil was observable already at the lowest concentration of 100 µg/mL which reduced about 50% of trophozoites after 144 h^54^ (Table II).

Preliminary study of the aqueous extract of the root from *Scutellaria baicalensis* which is an important herb in traditional Chinese medicine, exhibited strong trophocidal effect of 50 mg/mL solution against *Acanthamoeba royreba*, and lower efficiency against *A. castellanii* and *A. polyphaga* ^55^ (Table I). Further thorough study demonstrated anti-*Acanthamoeba* effects of the most important phytochemicals from the extract. The highest amoebicidal activity exhibited wogonoside with 0.5 log reduction in viability of *A. castellanii* and oroxylin A with 0.4 log reduction in viability of *A. polyphaga*, after 4 h of exposure*.* The most effective combination was baicalein and oroxylin A with 0.65 and 0.50 log reduction in viability of *A. castellanii* and *A. polyphaga*, respectively, after 4 h of exposure^56^ (Table II).

## DISCUSSION

This review is an overview of publications focused on the anti-*Acanthamoeba* effects of the plant extracts, essential oils and phytochemicals obtained from the species of the family Lamiaceae. To the best of our knowledge, all the published papers were included.

The most effective biologically active compounds included in the extracts are probably phenols, as carvacrol and thymol, and terpene alcohols. However, the proportion of these compounds in various extracts frequently varies and for clinical use, qualitative and quantitative analysis would be needed. The essential oils represent also an interesting research area. The results showed that due to a high concentration of active compounds they are more effective than extracts.

Low toxicity of the Lamiaceae plant extracts to tissue cultures enhances their possible potential for clinical use. Various species of the family Lamiaceae exhibited antiparasitic, but also antineoplastic, antiviral, antifungal, and antibacterial activities.^57^ The research demonstrated also interesting trophocidal and cysticidal effects against acanthamoebae. Several publications are available in this topic where the susceptibility to extracts was tested on clinical isolates of only two species ― *A. castellanii* or *A. polyphaga*.^41^ ^,^ ^43^ ^,^ ^46^ ^,^ ^49^ ^,^ ^50^ ^,^ ^52^ ^,^ ^54^ Some publications were based even on the assays on exclusively non-pathogenic environmental strains.^44^ ^,^ ^47^ ^,^ ^51^ ^,^ ^53^ This may represent a problem, because pathogenic strains may react differently in the experiments and published results can not be automatically adopted for clinical strains without a critical perspective. Even the initially pathogenic strains in prolonged axenic cultures exhibit frequently attenuated virulence which should be reactivated shortly before experiments.^58^ Further research is needed with inclusion of several clinical isolates because of possible differences in their susceptibility. Subsequently, *in vivo* studies should follow to verify the activity in live systems.

In Conclusion

We focused on a review of published studies that used plant extracts, essential oils, and phytochemicals from Lamiaceae plants against *Acanthamoeba*. As a whole, this review supplies significant information regarding the herbal products with acanthamoebicidal activity which would be extremely helpful for experimental and clinical trials and herbal combination therapy investigations. It is also necessary to find their active components and identify potential toxic effects which would lead to producing new effective, well-tolerated and safe drugs for treating the *Acanthamoeba* infections.
